# SOTXTSTREAM: Density-based self-organizing clustering of text streams

**DOI:** 10.1371/journal.pone.0180543

**Published:** 2017-07-07

**Authors:** Avory C. Bryant, Krzysztof J. Cios

**Affiliations:** 1 Department of Computer Science, Virginia Commonwealth University, Richmond, VA, United States of America; 2 Naval Surface Warfare Center Dahlgren Division, US Navy, Dahlgren, VA, United States of America; 3 Institute of Theoretical and Applied Informatics, Polish Academy of Sciences, Gliwice, Poland; Bangladesh University of Engineering and Technology, BANGLADESH

## Abstract

A streaming data clustering algorithm is presented building upon the density-based self-organizing stream clustering algorithm *SOSTREAM*. Many density-based clustering algorithms are limited by their inability to identify clusters with heterogeneous density. *SOSTREAM* addresses this limitation through the use of local (nearest neighbor-based) density determinations. Additionally, many stream clustering algorithms use a two-phase clustering approach. In the first phase, a micro-clustering solution is maintained online, while in the second phase, the micro-clustering solution is clustered offline to produce a macro solution. By performing self-organization techniques on micro-clusters in the online phase, *SOSTREAM* is able to maintain a macro clustering solution in a single phase. Leveraging concepts from *SOSTREAM*, a new density-based self-organizing text stream clustering algorithm, *SOTXTSTREAM*, is presented that addresses several shortcomings of *SOSTREAM*. Gains in clustering performance of this new algorithm are demonstrated on several real-world text stream datasets.

## Introduction

A primary means for sharing information amongst people is through the production and consumption of text. This fact can be observed in one’s daily interactions with text-based information sources such as news articles, blog/micro-blog posts, websites, academic publications, search engine queries/results, email, and computer logs. A common theme amongst these information sources is that they are naturally observed as a sequence or stream of text-based objects (e.g., article, post, query, or email). Given their abundance and size, the analysis of text streams is an important problem with respect to the analysis of big data.

One such analysis, useful in the exploration of large unlabeled datasets, is cluster analysis. In addition to the text-based applications of document organization; topic extraction; and outlier detection, in a streaming setting cluster analysis can be applied to problems of change-point detection. Examples of applications include identifying emergent trends in Twitter posts [1–3] and user queries [4], identifying new and tracking existing news stories [2, 5–7], and identifying spam emails [8].

Traditional non-streaming clustering approaches focus on the offline analysis of static, unordered data (e.g., partitioning, hierarchical, density-based, model-based, and grid-based cluster analysis). Here data is assumed to be stationary as well as independently and identically distributed. However, with streaming data such assumptions may be invalidated due to the potential for concept drift. Concept drift can best be described with respect to supervised learning, where properties of the target variable change over time.

An in-depth description of concept drift is presented in [9] with respect to Bayesian decision theory. Assuming a categorical response variable, concept drift is defined as changes in the data’s class conditional probabilities and/or prior class probabilities. Thus, the posterior probability of some object belonging to some class may change over time. In such a setting one can view clustering as follows, first assume that data is produced from some generative model. For example, object and class label pairs drawn from the joint probability density distribution defined by the conditional and prior probability distributions. With respect to clustering, objects are presented without class labels. Here the goal of clustering can be viewed as grouping the objects into sets, clusters, which correlate to the grouping, sets, defined by the hidden class labels. With this in mind, concept drift may be described with respect to unsupervised learning, where properties of the generative model change over time.

In addition to the differences mentioned above, the learning step faces increased memory and processing restrictions not seen in the non-streaming environment. First, with respect to time, learning is restricted to the time frame of the stream, as at any stream time *t* the learner’s view of the stream is restricted to stream objects arriving at or before *t* (i.e., the learner cannot look ahead into the future). Second, a stream’s arrival rate acts as an upper bound on per-object learning time (i.e., objects must be processed at the rate at which they arrive). Third, as the size of a stream may be unbounded, at any time *t*, it is unfeasible to maintain all prior objects (i.e., previously observed objects must be discarded).

A solution to the above issues is the use of adaptive online single-pass clustering algorithms. Adaptive clustering algorithms have the ability to grow or shrink the number of recognized clusters (i.e., capture the dynamics of the stream). In online learning, learning is restricted to one object at a time with an updated model being available after every object. Finally, a single-pass algorithm performs a single-pass over all objects never revisiting an object twice. An example of such a clustering algorithm is the Leader-Follower Clustering Algorithm (*LFCA*) [10, 11] which represents a greedy approach to the problem. A popular stream clustering approach, that trades-off between the benefits of online versus offline learning, is the *CLUSTREAM* algorithm [12]. Here online clustering is performed at a micro level. This micro solution at any time can be passed to an offline clustering step; this step producing a macro solution by clustering the micro solution.

In *LFCA*, summary representations of clusters (e.g., statistics such as centroids) are maintained online following the arrival of each new stream object. Here each new object is inserted into its nearest existing cluster assuming some insertion criterion is met. An insertion effectively updates the nearest cluster’s state (e.g., its cluster centroid is adjusted in the direction of the new object, and its weight increased) where the insertion criterion is associated with some distance-based threshold. If the insertion criterion is not met, a new singleton (single object) cluster is created from the new object. In either case, the object is immediately discarded and model updated. This last point leads to an important property of cluster summary representations; namely that they be incrementally updateable (i.e., without having to access all past inserted objects). Generally, the effect of such an update is relative to the current weight of the cluster that is also subject to some process of decay. In addition to this insertion process, several other cluster maintenance operations may be performed such as the deletion of old clusters; merging of near clusters; and splitting of large, disperse clusters. Examples of *LFCA* stream clustering algorithms include *CLUSTREAM*, *DENSTREAM* [13], *STREAMOPTICS* [14], *MRSTREAM* [15], *CLUSTREE* [16], *SOSTREAM* [17], *HASTREAM* [18, 19], and *SOTXTSTREAM*.

Three of the above density-based approaches are designed to handle clusters of heterogeneous density: *STREAMOPTICS*, *MRSTREAM*, and *HASTREAM*. *STREAMOPTICS* is a method for visualizing streams and is similar to the non-streaming density-based *OPTICS* [20]. *MRSTREAM* uses a grid-based clustering approach used to model data at multiple resolutions (i.e., densities). Unfortunately, such an approach is not well suited given high-dimensional data. *HASTREAM*, another hierarchical approach, maintains a density-based minimum spanning tree of clusters, where an offline clustering is produced via hierarchical edge cutting (see *HDBSCAN* [21]). *HASTREAM* maintains micro-clusters online using the *DENSTREAM* or *CLUSTREE* methods (i.e., this approach is primarily focused on the offline phase).

In regards to the above *LFCA* stream clustering algorithm, *SOSTREAM* is unique with respect to its use of self-organizing concepts. In *SOSTREAM*, the nearest cluster is updated by the new object, whereas its nearest neighbors are updated by the nearest cluster (i.e., this learning approach is similar to updating performed in Self-Organizing Maps (SOM) [22]). As with *LFCA*, the winning cluster and its neighborhood are updated if and only if some insertion criterion is meet (e.g., the distance between the nearest cluster and the new object is below or equal to some distance threshold). For *SOSTREAM*, this distance threshold is set to the distance between the nearest cluster and its *k*^*th*^-nearest neighbor (i.e., the distance threshold is dynamic and cluster-dependent). Finally, the winning cluster’s neighborhood is examined for potential mergers eliminating the need for performing a separate offline clustering step.

This last point represents the primary motivation behind the *SOTXTSTREAM* and *SOSTREAM* algorithms, which is the elimination of the offline clustering step required to produce a macro clustering solution. In both cases, this is achieved by effectively reducing the number of micro-clusters in the online phase via a *SOM*-like approach. With this in mind, the main contributions of *SOTXTSTREAM* correspond to improvements to the *SOSTREAM* algorithm for clustering streaming text, which include:

- Redesign of the algorithm with respect to the use of Cosine distance, as opposed to Euclidean, which is more appropriate for computing distances between documents.
- Redesign of the algorithm to effectively, with respect to performance, reduce the number of micro-cluster produced.
- Evaluation performed on several real-world disparate text stream with synthetic concept drift.

The remainder of this paper is structured as follows: prior work in clustering streaming text is presented in Background, *SOTXTSTREAM* is introduced in Materials and Methods, performance of *SOTXTSTREAM* is evaluated in Results and Discussion, and findings summarized in Conclusion.

## Background

Here prior work focusing on the use of online clustering approaches for the analysis of text is presented. Note the generic use of the term object, referring to a stream datum observation, is dropped in favor of document.

In [1, 4], the *IncrementalDBSCAN* [23] clustering algorithm is used to maintain an online *DBSCAN* [24] clustering solution on a sliding window of stream documents (user queries [4] and Twitter tweets [1]). This approach relies on the fact that the *DBSCAN* algorithm clusters data by local neighborhood observations. Specifically, it is assumed that the insertion or removal of a document has a local affect on the clustering solution. Unique aspects of the two approaches includes leveraging of click-through information [4], the use of a temporal penalty function [1], and the use of geographic information [1].

Online variants of the *kMEANS* clustering algorithm [8, 25, 26] have been applied to cluster document streams (websites [25], email [8], and Twitter tweets [26]). While [25] is a multi-pass iterative clustering approach, operating on stream segments, it does perform fading which is characteristic of online approaches. Specifically, a fading learning rate is applied at each iteration of *kMEANS* such that clusters are faded across segments. Concepts from *kMEANS++* [27], a non-random seeding *kMEANS* algorithm that guarantees an approximate solution, are incorporated into a stream clustering algorithm in [8]. Here a merge-and-reduce technique is used to maintain a set of core-sets, document set summaries, representing an approximate solution to a *kMEANS++* seeding (i.e., this is actually a solution to the *kMEDIODS* problem). In [26], an approximate kernel matrix of the stream is maintained using importance sampling where clustering is applied to the eigen decomposition of said matrix (i.e., kernel-based *kMEANS*).

Numerous examples of the online processing of text streams can be seen in work on topic detection and tracking [2, 5–7] focusing on streaming news articles. In these works, the main applications are first story detection and tracking. Similar to *LFCA*, first nearest neighbor classification is used where new documents are compared directly to previously observed documents. Here cluster membership of documents are maintained, as opposed to cluster summaries, where new documents are assigned to the cluster of their nearest prior document or assigned to a new cluster. Unique aspects of this work includes the use time-dependent document distances [5–7], and normalizing distances given some set of labeled documents [6, 7]. Additionally, [7] is unique in its use of text distances based on the minimum distance between overlapping text segments.

A computational bottleneck of *LFCA* lies in its solution to the *k*-nearest neighbor problem. An approximate solution to the *k*-nearest neighbor problem for high-dimensional data is Locality Sensitive Hashing LSH [28]. LSH hashes observations into bins such that similar observations are more likely to be hashed into the same bin (i.e., similar observations will have the same hash value with high probability whereas dissimilar observations will have the same hash value with low probability). In this way the complexity of identifying similar or near neighbors is reduced by limiting searches to the set of observations within the same bin. In [2] first nearest neighbor classification of documents is performed using the random projections method of LSH [29], adapted for the Cosine distance. Here a constant number of prior documents is maintained by limiting the number of documents assigned to each bin. This maintenance is performed by the removal of older documents in overflowing bins. Similarly, in [30], LSH is used with *LFCA* on a stream of XML documents. Here XML documents and their clusters are maintained as graphs where bloom filters are used to optimize set-based distance calculations. *LFCA* is performed on the XML graphs using the min-wise independent permutations method of LSH [31], adapted for the Jaccard distance.

Given their popular usage in text modeling, there exists prior work in online topic models as seen in [32, 33] for text streams. In [32], online topic-models are investigated for several topic models including von Mises-Fisher, Dirichlet Compound Multinomial, and Latent Dirichlet Allocation models. All approaches assume some initial model, where model updating procedures are presented for the insertion of new documents. In addition to the online topic models, an online-offline process is introduced that maintains the topic model online, periodically optimizing said model with an offline step (e.g., Gibbs sampling for Latent Dirichlet Allocation) using a set of previously observed documents. In [33] a multinomial mixture model of terms is combined with a translation model, used to model the relationship between terms and phrases, and fading model that discounts the effect of older documents. Here the topic model is maintained online by *LFCA* using summary statistics required to maintain a multinomial for each topic.

In [34], a *LFCA* stream clustering algorithm is presented for text and categorical data. This approach is novel with respect to the maintained cluster statistics, and includes sparse representations of weighted non-zero co-occurrence counts for terms. A similar approach is seen in [35] that combines social network and text-based distances into a single distance measure. Non-document clustering solutions to the problem of event detection in text streams are seen in [3, 36]. An offline approach to identifying emergent topics is presented in [3] by the identification and clustering of emergent terms in stream segments. This approach also incorporates social-network information (i.e., Twitter data) to detect emergent topics. In [36], the problem being investigated is that of maintaining frequent itemsets over a sliding window of stream instances with offline clustering. Lastly, in [37, 38] the focus is on maintaining dense components of a streaming term co-occurrence graph (i.e., graph-based approaches).

An important pre/online processing step relevant to the performance of document clustering is that of term (feature) weighting. Term weighting relies on some statistical knowledge of term usage in a document collection. However, in the streaming setting, term usage statistics may be unknown, incomplete, or subject to drift. This problem is not considered in this work, as online methodologies are compared with several offline ones (i.e., non-streaming clustering). Still, a review of potential solutions is presented below.

In [39], it is shown that some representative background corpus can be used for Term Frequency—Inverse Document Frequency (*TF-IDF*) weighting with a negligible effect on performance. Similarly, in [40], *incrementalTF-IDF*, continuously updating of term usage statistics, is shown to be effective given a sufficiently large set of initial documents.

Term weighting solutions [41–43] focus on weighting terms by their arrival rate in the stream (i.e., positively correlating term arrival rate with significance). Offline approaches presented in [41] and [43] use a popular method of modeling term burstiness by arrival rate [44], and by segmenting the stream and modeling expected random segment term counts using a binomial distribution. An online approach is presented in [42] by maintaining incremental means of term arrival rates. Similarly, [45] addresses the problem of maintaining online approximate frequent item counts, under polynomial decay, in data streams, though their focus is not on text.

Finally, supervised approaches such as [7, 46] perform term weighting assuming some known categorization of the documents. In [46], categories are assumed to represent separate network news text streams where significant terms are those that are highly weighted across many networks. Conversely, in [7], categories represent topics where a term’s weight is increased if it occurs in a small number of topics.

## Materials and methods

### Definitions

In this section definitions are presented for the required elements of the *SOTXTSTREAM* algorithm, summarized in Table 1.

**Table 1:** *SOTXTSTREAM* functions and parameters.

***X***	stream of text documents
**x**_*i*_	*i*th arriving document, *TF-IDF* weighted vector of document ***x*** dependent on term usage statistics of background collection ***B*** (see Eq (1)), of ***X***
*t*(***x***)	time stamp of document ***x***
*norm*(***x***)	normalized vector of ***x*** (see Eq (2))
*dist*(***a***, ***b***)	cosine distance between vectors ***a*** and ***b*** (see Eq (3))
*N*_*k*_(***a***, ***A***)	*k*-nearest neighbor function that returns the *k*-nearest neighbors of ***a*** in set ***A***. Assumes that the returned set is in ascending order with respect to distance from ***a***
*f*(Δ*t*)	function returns a fade value with respect to change in time (see Eq (4))
*λ*	controls the degree of fading in function *f* with respect to change in time
***M***	set of micro-clusters
***m***	micro-cluster in ***M*** defined by the features ***m***_***s***_ (linear sum), ***m***_*w*_ (weight), ***m***_*t*_0__ (update time), and ***m***_***c***_ (centroid)
*init*(***x***)	function initializes a singleton micro-cluster with document ***x*** (see Eq (5))
*insert*(***m***, ***x***)	function inserts document ***x*** into micro-cluster ***x*** (see Eq (6)
*fade*(***m***)	function fades micro-cluster ***m*** with respect to the current stream time (see Eq (7)
*merge*(***m***, ***m***′)	function creates a new micro-cluster by merging two existing micro-clusters ***m*** and ***m***′ (see Eq (8)
*adjust*(***m***, ***x***, *r*)	function adjusts micro-cluster ***m*** towards document ***x*** with respect to radius *r* (see Eq (10)
*β*(***x***, ***m***, *r*)	function returns the influence of document ***x*** on micro-cluster ***m*** given radius *r* (see Eq (11)
*m*_*thresh*_	micro-cluster merge threshold

Let ***X*** = 〈***x***_0_, …, ***x***_*i*_, …〉 define a continuous stream of text documents, such that for all documents ***x***_*i*_, *i* = 0…|***X***| − 1, index *i* indicates stream arrival order. Note that at any index *i*, all documents in the stream with index ***X***_≤*i*_ have been observed, whereas documents with index ***X***_>*i*_ have yet to be observed. Additionally, let function *t* define a time-stamp function $t:x_{i}\to Z_{\geq 0}|\forall x_{i}:t\left(x_{i}\right)\leq t\left(x_{i+1}\right)$ that maps stream documents to their time of arrival represented as an integer offset from the start of the stream, initialized to 0 (i.e. *t*(***x***_0_) = 0). While time-stamp function *t* allows one to define time epochs in which several or no stream documents arrive, for simplicity, here it is assumed that *t*(***x***_*i*_) = *i*.

For each stream document, let ***x***_*i*_ represent a term-frequency vector of length *d* such that $x_{i}\in Z_{\geq 0}^{d}$, and ***x***_*i*, *j*_, *j* = 0…*d* − 1, is the frequency of term *j* in document *i*. Furthermore, assume the existence of some background document collection ***B*** where ***B***^*j*^ = |{***b*** ∈ ***B*** ∣***b***_*j*_ > 0}| is the number of documents in ***B*** containing term *j*. Let function *tfidf*(***x***_*i*_, *j*, ***B***) return the *TF-IDF* weighted value of term *j* in document ***x***_*i*_ given background corpus ***B***:

$$\begin{array}{c} tfidf\left(x_{i},j,B\right)=x_{i,j}\times \log\frac{\left|B\right|}{B^{j}}, \end{array}$$ (1)

where $tfidf\left(x_{i},j,B\right)\in R_{\geq 0}^{d}$. For the remainder of this paper, all references to stream documents, say ***x***, refer to the *TF-IDF* weighted vector of ***x***, ***x***_*j* = 0…*d*−1_ = *tfidf*(***x***, *j*, ***B***), not the term-frequency vector.

For any vector $x\in R_{\geq 0}^{d}$, normalize function *norm* returns the normalized vector of ***x***:

$$\begin{array}{c} norm\left(x\right)=\frac{x}{\left||x|\right|} \end{array}$$ (2)

where $norm\left(x\right)\in R_{\geq 0}^{d}$ and ||*norm*(***x***)|| = 1.

For any two vectors $x,y\in R_{\geq 0}^{d}$, distance function *dist* returns the distance between ***x*** and ***y***. Here function *dist* is defined using cosine distance:

$$\begin{array}{c} dist\left(x,y\right)=1-\frac{x\cdot y}{\left||x||||y|\right|} \end{array}$$ (3)

where *dist*(***x***, ***y***)∈[0, 1].

Given a set of vectors ***Y***, positive integer *k*, and vector ***x***, let function *N*_*k*_(***x***, ***Y***) return the set of *k* nearest neighbors, defined by *dist*, of ***x*** in ***Y***. Assume that nearest neighbors in *N*_*k*_(***x***, ***Y***) are returned in ascending order according to their distance from ***x***, such that first index of the returned set is the nearest instance in ***Y*** from ***x***.

Stream ***X*** is modeled by maintaining a set of micro-clusters ***M*** whose state prior to observing document ***x***_*i*_ is dependent on the previously observed *i* − 1 documents, ***X***_<*i*_. At document ***x***_*i*_, each micro-cluster ***m*** ∈ ***M*** represents a subset of documents, ***m*** ⊆ ***X***_<*i*_, where ***M*** represents a clustering of ***X***_<*i*_ such that ⋃_***m*** ∈ ***M***_ ***m*** = ***X***_<*i*_ and ∀***m***, ***m***′ ∈ ***M*** where ***m*** ≠ ***m***′, ***m***∩***m***′ = ∅. The set of documents in micro-cluster ***m*** define its summary representation, a time-dependent weight and centroid, using the fading function:

$$\begin{array}{c} f\left(\Delta t\right)=2^{-\lambda \Delta t} \end{array}$$ (4)

Note that this assumes that each document contributes a weight of one to the model at insertion (i.e., at Δ*t* = 0). Micro-cluster based clustering can be attributed to the *BIRCH* [47] algorithm, with a faded variant for streaming introduced in *CLUSTREAM* [12]. The following micro-cluster definition, insertion, and fading schemes are similar to the *CLUSTREAM* approach.

**Definition 1 (Micro-Cluster)** For a subset of documents ***Y*** ⊆ ***X***_<*i*_, *micro-cluster* ***m*** at stream time *t* = *t*(***x***_*i*_) is defined by the triple 〈***s***, *w*, *t*_0_〉. Here *w* is the micro-cluster’s weight, *w* = ∑_***y*** ∈ ***Y***_ *f*(*t* − *t*(***y***)); ***s*** the weighted linear sum of the normalized *TF-IDF* weighted documents in ***Y***, ***s*** = ∑_***y*** ∈ ***Y***_ *f*(*t* − *t*(***y***)) × *norm*(***y***); and *t*_0_ the time at which the micro-cluster was last updated, *t*_0_ = max_***y*** ∈ ***Y***_ *t*(***y***). Additionally, let ***c*** be the centroid of ***m*** defined as ***c*** = ***s***/*w*.

Any document ***x*** can be used to initialize a singleton micro-cluster ***m*** according to the function *init* as follows:

$$\begin{array}{c} init(x)=\langle norm(x),1,t(x)\rangle \end{array}$$ (5)

Note that in Def 1 it is assumed that the set of all previously observed documents, ***X***_<*i*_, is maintained throughout the stream; an impractical assumption as ***X*** may be unbounded. Fortunately, the summarizing variables of each micro-cluster, 〈***s***, *w*, *t*_0_〉, can be updated incrementally at the insertion of each stream document (see [12]). Consider the insertion of stream document ***x***_*i*_ with time stamp *t* = *t*(***x***_*i*_) into some micro-cluster ***m***. In this case, ***m*** can be updated by fading ***m***’s variables before incrementing it with document ***x***_*i*_ as seen in function *insert*:

$$\begin{array}{c} insert\left(m,x_{i}\right)=\left\langle f\left(t-m_{t_{0}}\right)\times m_{s}+norm\left(x_{i}\right),f\left(t-m_{t_{0}}\right)\times m_{w}+1,t\right\rangle \end{array}$$ (6)

Likewise, for any unaffected micro-cluster ***m***′ ≠ ***m*** at time stamp *t*, ***m***′ can be faded without insertion according to the function *fade*:

$$\begin{array}{c} fade\left(m^{\prime }\right)=\left\langle f\left(t-m_{t_{0}}^{\prime }\right)\times m_{s}^{\prime },f\left(t-m_{t_{0}}^{\prime }\right)\times m_{w}^{\prime },t\right\rangle \end{array}$$ (7)

Any pair of micro-clusters ***m*** and ***m***′ can be merged to create a new micro-cluster. This is achieved by the fading and addition of their variables as seen in function *merge*:

$$\begin{array}{ccc} merge(m,m^{\prime }) & = & \langle f\left(t-m_{t_{0}}\right)\times m_{s}+f\left(t-m_{t_{0}}^{\prime }\right)\times m_{s}^{\prime },\\ & & \;\;f\left(t-m_{t_{0}}\right)\times m_{w}+f\left(t-m_{t_{0}}^{\prime }\right)\times m_{w}^{\prime },t\rangle \end{array}$$ (8)

Recall that the *SOM* algorithm [22] is used to produce a lower dimensional representation of a dataset by mapping instances onto a grid of nodes (e.g., a 2-dimensional square grid). This mapping is obtained by learning a vector of weights, of the same dimension as the instances in the dataset, for each node, that are used to map instances onto the grid (i.e., to the closest node given the distance between a nodes weight vector and an instance). Node weight learning is performed over a series of learning steps (batch observation of the dataset) where for a given dataset ***X***, at each next step *s* + 1 the weight vector ***W***_***v***_(*s* + 1) of node ***v*** is updated as follows:

$$\begin{array}{c} W_{v}\left(s+1\right)=\sum_{x\in X}W_{v}\left(s\right)+\theta \left(u,v,s\right)\alpha \left(s\right)\left(x-W_{v}\left(s\right)\right) \end{array}$$ (9)

where function *α* is the learning rate (monotonically decreasing with respect to *s*), ***u*** is the closest node to ***x*** (according to the distance between ***x*** and the weight vector of node ***u***), and *θ* is a neighborhood function that returns the distance from ***u*** to ***v*** at step *s* (e.g, a Gaussian function centered at ***u*** with monotonically decreasing variance with respect to step *s*). Note that the distance returned by the neighborhood function *θ* is not related to node weight vectors, but rather the location of nodes on the grid.

Similar to the concept of updating neighbors of the winning node in *SOM*, when inserting stream document ***x***_*i*_ at time *t* = *t*(***x***_*i*_) into winning micro-cluster ***m***, some neighboring micro-cluster ***m***′ may likewise be updated, adjusted, by the insertion. Neighboring micro-cluster ***m***′ can be adjusted, non-insertion, by ***x***_*i*_ according to function *adjust* defined as:

$$\begin{array}{ccc} adjust(m^{\prime },x_{i},r) & = & \langle f\left(t-m_{t_{0}}^{\prime }\right)\times m_{s}^{\prime }+\beta \left(x_{i},m^{\prime },r\right)\times norm\left(x_{i}\right),\\ & & \;\;f\left(t-m_{t_{0}}^{\prime }\right)\times m_{w}^{\prime }+\beta \left(x_{i},m^{\prime },r\right),t\rangle . \end{array}$$ (10)

where function *β* defines the degree of influence, weight of the adjustment, the insertion of ***x***_*i*_ has on neighboring micro-cluster ***m***′ given some radius *r* (0 ≤ *r* ≤ 1).

$$\begin{array}{c} \beta \left(x_{i},m^{\prime },r\right)=e^{-\frac{dist(x_{i},m^{\prime })}{2r^{2}}} \end{array}$$ (11)

Note that influence function *β* is dependent on the distance from ***m***′ to ***x***_*i*_ and radius *r*. Specifically, given a fixed radius, function *beta* is monotonically decreasing with respect to this distance. Also note that 0 ≤ *β*(***x***_*i*_, ***m***′, *r*)≤1 as 0 ≤ *dist*(***x***_*i*_, ***m***′) ≤ 1.

In contrast to *SOM*, in *SOTXTSTREAM* a dynamic set of micro-clusters is updated (as opposed to a grid of nodes) at the arrival of each new document (as opposed to batch observation of the entire dataset). Additionally, updating is limited to the new document’s nearest micro-cluster (Eq (6)), and some neighboring set of micro-clusters (Eq (10)). Furthermore, in *SOTXTSTREAM*, Eq (11) represents the learning weight expressed by the product *θ*(***u***, ***v***, *s*)*α*(*s*) in Eq (9) where *θ* is a Gaussian function. Finally, while *SOM* (Eq (9)) updates nodes (micro-clusters) by a signed difference, the update in *SOTXTSTREAM* (Eq (10)) is equivalent to an online mean with respect to the weight of a micro-cluster.

### Stream clustering algorithm

**Fig 1:**
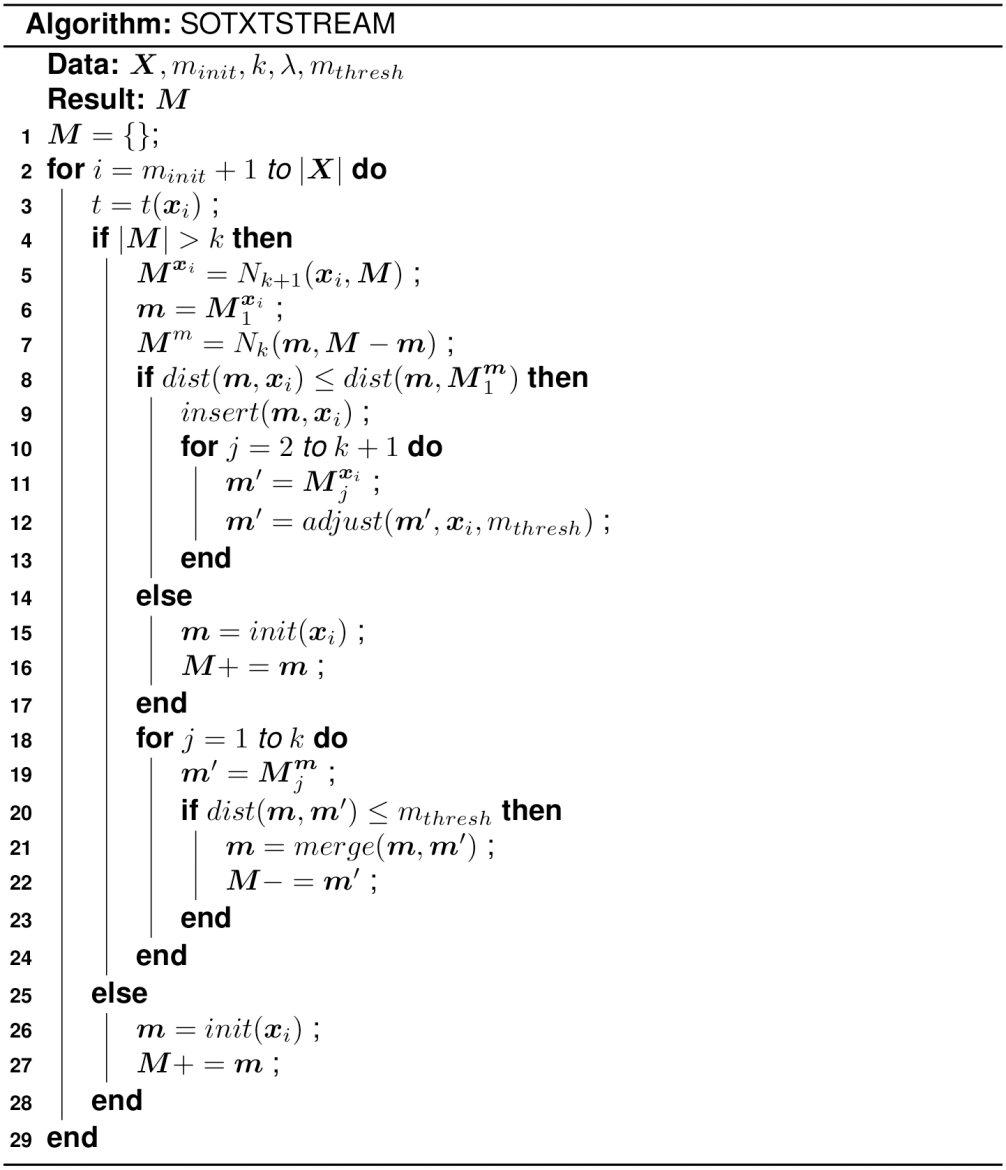
Pseudo-code for the *SOTXTSTREAM* algorithm.

In this section the *SOTXTSTREAM* clustering algorithm (Fig 1) is described. Beginning with some document stream ***X*** and empty set of micro-clusters ***M***, for next stream document ***x***_*i*_, if the current number of micro-clusters is less than or equal to *k* than a new singleton micro-cluster is created for the new document (Eq (5)) and inserted into ***M***. This is a necessary requirement as the algorithm requires at least *k* micro-clusters to form a *k*-nearest neighborhood. Note that the set of micro-clusters ***M*** is initialized with singleton micro-clusters (Eq (5)) for the first *k* + 1 documents (i.e., after initialization |***M***| = *k* + 1 with the next document occurring at index *k* + 2). For small values of *k*, and perhaps general, one may consider initializing the set of micro-clusters to some fixed number of initial stream documents. However, though not reported here, such an initialization has shown to have a negligible impact on clustering performance in our experimentation.

If the number of micro-clusters is greater than *k*, than the *k* + 1 nearest neighborhood $M^{x_{i}}\subseteq M$ for stream document ***x***_*i*_ is found along with the *k* nearest neighbor ***M***^***m***^ ∈ ***M*** of ***x***_*i*_’s nearest micro-cluster $m=M_{1}^{x_{i}}$. Note that the distance between a stream document ***x*** and micro-cluster ***m*** is calculated between document vector ***x*** and micro-cluster centroid vector ***m***_*c*_. New document, ***x***_*i*_, is inserted into its nearest micro-cluster (Eq (6)), ***m***, if the distance from ***x***_*i*_ to ***m*** is less than or equal to the distance between ***m*** and its nearest micro-cluster in ***M***. As with a violation of the size criteria on ***M***, if ***x***_*i*_ is not inserted into ***m***, then ***x***_*i*_ is used to create a singleton micro-cluster (Eq (5)) which is inserted into ***M***.

Next, if ***x***_*i*_ was inserted into its nearest micro-cluster ***m***, then ***x***_*i*_’s remaining *k* nearest neighbor micro-clusters ($M^{x_{i}}-m$) are adjusted towards ***x***_*i*_ (Eq (10)). Radius *r* of the influence function (Eq (11)) is set to the merge threshold *m*_*thresh*_. Note that such an approach represents a weighted competitive learning approach. Here self-organizing is dependent on the degree of intersections between the two *k*-nearest micro-cluster sets of ***m*** and ***x***_*i*_. Finally, the nearest micro-cluster ***m*** is merged with its *k*-nearest neighbors if the distance between them is less than or equal to merge threshold *m*_*thresh*_ (Eq (8).

Though not addressed here, a common step in micro-cluster based *LFCA* approaches, such as *SOTXTSTREAM*, is the periodic deletion of aging micro-clusters by a minimum weight threshold. For evaluation purposes, this step was not performed, though the algorithm outlined in Fig 1 could be easily modified to perform deletion (e.g., using the previously defined *fade* function (Eq (8)).

### Other stream clustering algorithms

In this section we describe two stream clustering algorithms that are used to evaluate the performance of *SOTXTSTREAM* in Results and Discussion. *SOSTREAM* which *SOTXTSTREAM* builds upon, and a basic *LFCA*-based stream clustering algorithm which we refer to as *LSTREAM*. *LSTREAM* is most related to the prior work presented on topic detection and tracking [2, 5–7], and may be viewed as a simple baseline with respect to micro-cluster approaches [12–19].

Most importantly, like *SOTXTSTREAM*, these approaches require a single online phase to produce a macro clustering solution via the merging of micro-clusters. Whereas most other micro-cluster approaches require an additional offline clustering phase. For this reason we limited our analysis to the listed approaches.

#### SOSTREAM

Two versions of *SOSTREAM* are present in [17], corresponding to versions with and without fading. The fading version can be interpreted as being equivalent to *SOTXTSTREAM* with respect to initialization Eq (5), insertion Eq (6), fading Eq (7), and merging Eq (8) of micro-clusters.

A micro-cluster in *SOSTREAM* is defined by the triple <***c***, *n*, *r*> representing a micro-cluster’s centroid, weight, and radius. Note that equivalent insertion and merging functions for centroid ***c*** can be defined with respect to weight *n*, faded according to Eq (7). For example, the centroid of micro-cluster ***m***, ***m***_***c***_, can be updated by inserting stream document ***x*** as ***m***_***c***_ = (***m***_*n*_ × ***m***_***c***_+***x***)/(***m***_*n*_+1). Similarily, the centroid of micro-cluster ***m*** can be merged with the centroid of some other micro-cluster ***m***′ by $\left(m_{n}\times m_{c}+m_{n}^{\prime }\times m_{c}^{\prime }\right)/\left(m_{n}+m_{n}^{\prime }\right)$. Radius *r* of micro-cluster ***m*** is initialized to 0 and updated at insertions into ***m***. This update sets the value of *r* to the distance from ***m*** to its *k*-nearest neighbor in the set of current micro-clusters ***M***, $r=dist(m,M_{k}^{m})$ where ***M***^***m***^ = *N*_*k*_(***m***, ***M*** − ***m***).

Similar to Eq (10), when inserting stream document ***x*** into winning micro-cluster ***m*** some neighboring micro-cluster ***m***′ of ***m*** may likewise be updated, adjusted, by the insertion. The centroid of neighboring micro-cluster ***m***′, $m_{c}^{\prime }$, is adjusted by ***m*** as follows:

$$\begin{array}{c} m_{c}^{\prime }=m_{c}^{\prime }+\alpha \times \beta \left(m_{c},m_{c}^{\prime },m_{r}\right)\times \left(m_{c}-m_{c}^{\prime }\right) \end{array}$$ (12)

where *α* is a learning rate (0 ≤ *α* ≤ 1), ***m***_*r*_ the radius of ***m***, and *β* the influence function as defined in Eq (11). Differences between Eqs (10) and (12) are discussed below within the context of the streaming algorithm.

*SOSTREAM* follows the streaming algorithm outlined in Fig 1 with several key differences. First, in *SOSTREAM*, document ***x***_*i*_ is inserted into its nearest micro-cluster, ***m***, if the distance from ***x***_*i*_ to ***m*** is less than or equal to the distance between ***m*** and its *k*-nearest neighbor micro-cluster in ***M***. Recall from Fig 1, in *SOTXTSTREAM*, this insertion threshold is set to the distance between ***m*** and its nearest neighbor in ***M***. Several factors contributed to the choice of the latter approach. Primarily, use of the nearest neighbor decouples the use of *k* in the insertion decision from its use in the neighborhood adjusting and merging processes. With respect to *SOSTREAM*, this dependence results in a preference towards solutions with smaller values of *k*, which limits the effect of the adjusting and merging phases.

Second, in *SOSTREAM*, if ***x***_*i*_ is inserted into its nearest micro-cluster ***m***, then ***m***’s *k*-nearest neighbors in ***M*** are adjusted towards ***m*** (Eqs (12) and (11)). Recall from Fig 1, in *SOTXTSTREAM*, ***x***_*i*_’s remaining *k*-nearest neighbor micro-clusters ($M^{x_{i}}-m$) are adjusted towards ***x***_*i*_ (Eqs (10) and (11)). The latter approach is selected for several reasons. Adjusting towards the new document ***x***_*i*_, as opposed to its nearest micro-cluster ***m*** is more similar to the original *SOM* approach. Additionally, while it seems more appropriate to update ***m***’s nearest neighbors with respect to *SOM*; the use of the cosine distance confounds such an approach. Specifically, as cosine distance does not ensure the triangle inequality, closeness to ***x***_*i*_’s nearest neighbor ***m*** does not guarantee closeness to ***x***_*i*_. In addition to this last point, recall in *SOM* that a node’s neighborhood is determined with respect to the node grid structure. As no such grid structure exists here, limiting updates to neighbors of ***m*** (as opposed to ***x***_*i*_) seemed inappropriate.

Other differences in the adjustment of neighboring micro-clusters, observed in Eqs (10) and (12), include the following. First, Eq (12) updates a micro-cluster by a signed difference, while Eq (10) is equivalent to an online mean with respect to the weight of a micro-cluster. The latter approach being more appropriate for micro-clusters representing document centroids where it is assumed that centroid $c\in R_{\geq 0}^{d}$. Second, in Eq (12), the effect of an adjustment on a micro-cluster’s centroid is independent of the micro-cluster’s size, whereas in Eq (10) the effect is relative to the micro-cluster’s weight (i.e., the larger the weight, the smaller the impact and vice versa). This requires the use of an additional parameter, *α*, in Eq (12) to reduce the effect of the adjustment. Third, in Eq (12), the radius of the influence function Eq (11)) is set to the radius of ***m***, ***m***_*r*_, which is the distance between ***m*** and its *k* nearest neighbor in ***M***. Recall from Fig 1, in *SOTXTSTREAM*, the radius of the influence function is set to the merge threshold *m*_*thresh*_. This latter approach is chosen due to the relationship between the fading and merging processes. Specifically, as the merge threshold effectively defines a minimum distance between micro-clusters, its use in defining the impact a new stream document has on neighboring micro-clusters seemed appropriate.

Finally, in *SOSTREAM*, the merging of neighboring micro-clusters, as seen in Fig 1, has the addition requirement (i.e., in addition to the distance threshold) that the area of the micro-clusters, defined by their radii, must be overlapping. Note that this makes the use of a merge threshold optional in *SOSTREAM* where the overlapping criterion might be deemed sufficient. However, it has been observed that the performance of *SOSTREAM* is highly dependent on the use of a merge threshold. Similarly, though not reported here, our experiments indicate that the use of the overlapping criterion has a negligible effect on performance while using a merge threshold.

#### LSTREAM

To simplify the description of *LSTREAM* along with the interpretation of its results, *SOTXTSTREAM*’s micro-cluster definition Def 1 along with its initialization Eq (5), insertion Eq (6), fading Eq (7), and merging Eq (8) functions are reused in *LSTREAM*.

With respect to the stream clustering algorithm, *LSTREAM* requires a single distance-based threshold parameter, *d*_*thresh*_, and is outlined as follows. A new document is inserted into its nearest existing micro-cluster if their distance is less than or equal to *d*_*thresh*_. Otherwise a new micro-cluster is created for the new document. If the new document is inserted into an existing micro-cluster, then the updated micro-cluster is merged with any existing micro-clusters within *d*_*thresh*_ distance from it.

Note the performance of *LSTREAM*, with respect to *SOTXTSTREAM*, is of particular interest as it lacks the *SOM*-like adjustment phase while incorporating a more aggressive merging phase. Thus, the benefits of the adjustment phase in *SOTXTSTREAM* can be observed with respect to *LSTREAM*. In particular, the number of micro-cluster produced by each algorithm is of interest, along with their evaluation performance.

## Results and discussion

To evaluate the performance of *SOTXTSTREAM* several real-world text collections were used, and results compared with *SOSTREAM*, *kMEANS*, *LSTREAM*. *kMEANS* was chosen to contrast the performance of the streaming approaches with a popular non-streaming clustering algorithm. Synthetic versions of each collection were created to examine the performance of each algorithm given concept drift.

Note that Cosine distance was used in all of the algorithms, along with normalized *TF-IDF* weighted document.

### Experiment

Two methods were used to produce stream orderings for each text collections (i.e. the order in which documents arrive). First, a random ordering which is equivalent to sampling without replacement from the prior class distribution of the collection. Stream orderings of this type were considered to lack concept drift as they are dependent on the observed prior class distribution of the collection.

Second, a random ordering which is based on randomly generating the order in which classes arrive in the stream. Stream orderings of this type were considered to exhibit concept drift as the prior class distribution is dependent on the random class ordering and are highly dependent on the position of the stream. Streams of this second type are referred to as synthetic versions of the dataset.

Note that in the first random ordering, random sampling without replacement, sampling is not independent, but does satisfy exchangeability. In the case of the second random ordering, the classes are mutually exclusive within the stream, and exchangeability is no longer satisfied. In other words, all orderings are not equally likely as some orderings have zero probability due to the classes being mutually exclusive within the stream.

Performance results are reported as the average performance given 100 random orderings of the above two types for each dataset. In the case of *kMEANS* where the effects of data ordering are minimal, a single ordering was used. Note that documents are not evenly distributed across categories in all cases except for the *20newsgroups* collection.

Adjusted Rand Index (ARI) [48] was used to evaluate the performance of the clustering algorithms on each dataset. ARI is a similarity measure between two data clusterings that is adjusted for chance and is related to accuracy. For a fair comparison, optimal parameters with respect to ARI were discovered via grid search, at 10^−2^ precision, over a range of their values. Optimal parameters were chosen by the maximum average ARI performance over the 100 random orderings

#### Data

Five unique text datasets were selected for evaluation, representing a diverse sample of potential text streams (e.g., message posts, news articles, scientific publications, and email).

***20newsgroups* [49, 50]** Subset of the 20newsgroups collection, 9,595 documents from 10 categories, of message posts collected from various news groups. Documents were limited to the set of top 10 most distinct categories (see definition of distinct below).
***arxiv2015* [51]** Subset of the arXiv collection, 8424 documents from 40 categories, of scientific bibliographic publications limited to documents published in 2015. Documents labeled by multiple categories were discarded, and only documents from the remaining top 40 most distinct categories kept.
***ecue* [52]** Collection of 9,978 emails, categorized as spam or non-spam, collected from a single individual’s mailbox.
***reuters21578* [50, 53]** Subset of the Reuters21578 collection, 8,257 documents from 65 categories, of Reuters newswire articles. Documents labeled by multiple categories were discarded.
***tdt2* [50, 54]** Subset of the NIST Topic Detection and Tracking collection, 9,302 documents, of news documents collected from multiple sources. Documents labeled by multiple categories were discarded, and only documents from the remaining top 30 largest categories kept.
***syn20newsgroups*, *synarxiv2015*, *synreuters21578*, *syntdt2*** Synthetic versions of the *20newsgroups*, *arxiv2015*, *reuters21578*, and *tdt2* datasets generated by defining their document stream orderings as follows. For each dataset, categories were randomly ordered and the first three categories marked as active. Documents were then randomly drawn, without replacement, from the active categories until a category was exhausted of documents. At which point the next category in the category ordering was marked as active and the process continued until all categories were exhausted. Note that the *ecue* dataset was not included as it consisted of only two categories.

Here a category is defined as being distinct when the ratio of the category’s intra-document similarity versus its inter-document similarity is small (with respect to the ratios of all categories). For inter and intra-document similarity calculations, the average pair-wise document similarity was used. For two datasets, *20newsgroups* and *arxiv2015*, it was deemed necessary to limit analysis to the set of most distinct categories. In particular, this was due to the existence of hierarchical relationships within the categorizations (e.g., one category might be a child of another).

#### Data preprocessing

Recall that each document is represented as a normalized *TF-IDF* weighted vector of terms. In all cases, except for *arxiv2015*, datasets were obtained in the form of document term frequency vectors (i.e., no term tokenization or filtering was required). With respect to *arxiv2015*, the Lucene Letter tokenizer was used along with several existing Lucene filters (Standard, ASCIIFolding, Lowercase, Length (3), Stop (default list), and PorterStem). For each document collection, the number of terms was limited to the top 2000 selected by term document frequency. Additionally, for each document collection, term usage statistics for *TF-IDF* weighting were calculated using the entire collection (i.e., the actual collection was used as the background collection ***B*** in Eq (1)). Finally, documents consisting of fewer than 10 terms, not necessarily unique, were discarded. Note that the number of documents reported above is the remaining number of documents after applying all of the above filters. In all cases, the actual number of discarded documents due to term and document length filtering was minimal.

#### Parameters of clustering algorithms

Descriptions of each of the optimized parameter along with their range of possible values for each clustering approach are as follows:

***kMEANS*** Number of clusters 1 ≤ *k* ≤ 100.
***LSTREAM*** Distance threshold 0 ≤ *d*_*thresh*_ ≤ 1 for insertion and merging.
***SOSTREAM*** Number of nearest neighbors 1 ≤ *k* ≤ 20 for insertion, adjusting, and merging; constant learning rate 0 < *α* ≤ 1 for adjusting; and cluster merge threshold 0 < *m*_*thresh*_ < 1.
***SOTXTSTREAM*** Number of nearest neighbors 1 ≤ *k* ≤ 20 for adjusting and merging, and cluster merge threshold 0 < *m*_*thresh*_ < 1.

In addition to the above parameters, *SOSTREAM* and *SOTXTSTREAM* require a fading parameter *λ*. Given a dataset containing *n* documents, *λ* was set such that the weight of the first document at the end of the stream, *f*(*n*), is equal to $\frac{1}{n}$:

$$\begin{array}{c} \frac{1}{n}=2^{-\lambda n} \end{array}$$ (13)

Eq (13) can be rearranged to solve for *λ* as follows:

$$\begin{array}{c} \lambda =-\frac{\log_{2}\frac{1}{n}}{n} \end{array}$$ (14)

Note that in practice the value of this parameter would be set using domain knowledge or memory/computational constraints. For example, given a stream of news documents one may choose a *λ* that fades out old documents after a month. Optimal values for each algorithm-dataset pair are reported in Table 2.

**Table 2:** Clustering parameters.

Algorithm	Dataset				
*kMEANS*		***k***			
	*20newsgroups*	12			
	*arxiv2015*	14			
	*ecue*	3			
	*reuters21578*	3			
	*tdt2*	10			
*LSTREAM*		***d***_***thresh***_	**λ**		
	*20newsgroups*	0.68	0.001		
	*arxiv2015*	0.60	0.001		
	*ecue*	0.72	0.001		
	*reuters21578*	0.70	0.001		
	*tdt2*	0.65	0.001		
	*syn20newsgroups*	0.67	0.001		
	*synarxiv2015*	0.64	0.001		
	*synreuters21578*	0.71	0.001		
	*syntdt2*	0.66	0.001		
*SOSTREAM*		***k***	**α**	***m***_***thresh***_	**λ**
	*20newsgroups*	1	0.10	0.55	0.001
	*arxiv2015*	1	0.01	0.58	0.001
	*ecue*	1	0.31	0.50	0.001
	*reuters21578*	1	0.08	0.60	0.001
	*tdt2*	1	0.01	0.60	0.001
	*syn20newsgroups*	1	0.01	0.64	0.001
	*synarxiv2015*	1	0.01	0.60	0.001
	*synreuters21578*	1	0.10	0.59	0.001
	*syntdt2*	1	0.01	0.61	0.001
*SOTXTSTREAM*		***k***	***m***_***thresh***_	**λ**	
	*20newsgroups*	20	0.60	0.001	
	*arxiv2015*	5	0.54	0.001	
	*ecue*	8	0.56	0.001	
	*reuters21578*	17	0.65	0.001	
	*tdt2*	20	0.47	0.001	
	*syn20newsgroups*	15	0.58	0.001	
	*synarxiv2015*	18	0.52	0.001	
	*synreuters21578*	14	0.56	0.001	
	*syntdt2*	6	0.58	0.001	

### Results

Tables 3, 4 and 5 show the average ARI, Purity, and number of cluster results for each clustering method and evaluation dataset pair. Purity of a cluster is defined as the ratio of documents belonging to the majority category in a cluster, whereas Purity of a clustering is the weighted (by cluster size) average of cluster purity with respect to its clusters. As Purity is naturally biased towards solutions that produce a large amount of clusters, the discussion and conclusions are focused on ARI results. In all cases, ARI performance of *SOTXTSTREAM* outperforms or is equivalent to the performance of the other two streaming algorithms, *LSTREAM* and *SOSTREAM*. Additionally, *SOTXTSTREAM* outperforms *kMEANS*, by ARI, in four of the five non-synthetic datasets. ARI performance for *kMEANS* is not reported on the synthetic datasets as its performance is independent of stream ordering.

**Table 3:** Clustering performance by ARI.

Dataset	*kMEANS*	*LSTREAM*	*SOSTREAM*	*SOTXTSTREAM*
*20newsgroups*	0.66	0.39	0.25	**0.69**
*arxiv2015*	**0.60**	0.46	0.44	0.50
*ecue*	0.19	0.19	**0.23**	**0.23**
*reuters21578*	0.47	0.74	0.68	**0.94**
*tdt2*	0.70	0.77	0.70	**0.93**
*syn20newsgroups*	-	0.32	0.30	**0.47**
*synarxiv2015*	-	0.50	0.48	**0.81**
*synreuters21578*	-	0.45	0.58	**0.60**
*syntdt2*	-	0.74	0.65	**0.85**

**Table 4:** Clustering performance by purity.

Dataset	*kMEANS*	*LSTREAM*	*SOSTREAM*	*SOTXTSTREAM*
*20newsgroups*	0.78	0.76	0.69	0.79
*arxiv2015*	0.70	0.83	0.79	0.74
*ecue*	0.89	0.90	0.91	0.90
*reuters21578*	0.62	0.85	0.80	0.87
*tdt2*	0.73	0.94	0.91	0.97
*syn20newsgroups*	-	0.71	0.68	0.65
*synarxiv2015*	-	0.71	0.70	0.81
*synreuters21578*	-	0.73	0.76	0.74
*syntdt2*	-	0.92	0.87	0.92

**Table 5:** Number of clusters.

Dataset	*kMEANS*	*LSTREAM*	*SOSTREAM*	*SOTXTSTREAM*
*20newsgroups*	12	1226	1633	73
*arxiv2015*	14	2230	1637	750
*ecue*	3	228	315	60
*reuters21578*	3	852	1067	41
*tdt2*	10	957	1007	106
*syn20newsgroups*	-	1363	1179	154
*synarxiv2015*	-	1466	1336	121
*synreuters21578*	-	730	1101	122
*syntdt2*	-	931	1015	39

The poor overall performance on *ecue* can be attributed to the classification scheme of the data. Consider that documents are expected to cluster around topical similarities given the features and weighting scheme used (i.e., the distinction between spam and non-spam emails may not be entirely topical). In such a case, Purity is a more appropriate measure where results can be interpreted as the correlation between the topical categorization and some other categorization scheme (i.e, topical versus spam/non-spam). In fact, all algorithms perform relatively well on the *ecue* dataset with respect to Purity. In any case, there appears to be a clear correlation between a document’s topic and its being spam/non-span. Thus, poor ARI performance is undoubtedly due to the existence of numerous within-category topics.

With respect to number of clusters, *SOTXTSTREAM* produces far less clusters than the two other streaming algorithms, *LSTREAM* and *SOSTREAM*. This reduced number of clusters undoubtedly contributes to the overall superiority of *SOTXTSTREAM* with respect to ARI performance. Of course parameters could be selected for both *LSTREAM* and *SOSTREAM* to produce solutions which result in a smaller number of micro-clusters, though these solution would result in a decrease in ARI performance. In other words, neither solution can effectively, with respect to ARI performance, reduce the number of clusters as compared to *SOTXTSTREAM*.

To test the significance of the ARI performance results Wilcoxon signed-ranks tests [55] were used. This approach being suggested in [56] for comparing two classifiers over multiple datasets. Table 6 shows the resulting p-values from these tests, for each pair of clustering algorithms, which was applied to the ARI performance reported in Table 3. From these results, one can conclude that the difference between ARI performance of *SOTXTSTREAM* is significant with repect to the performance of both *LSTREAM* and *SOSTREAM*.

**Table 6:** Wilcoxon signed-ranks test p-values.

Algorithm	*LSTREAM*	*SOSTREAM*	*SOTXTSTREAM*
*kMEANS*	0.496	0.301	0.129
*LSTREAM*	-	0.250	**0.004**
*SOSTREAM*	-	-	**0.004**

**Fig 2:**
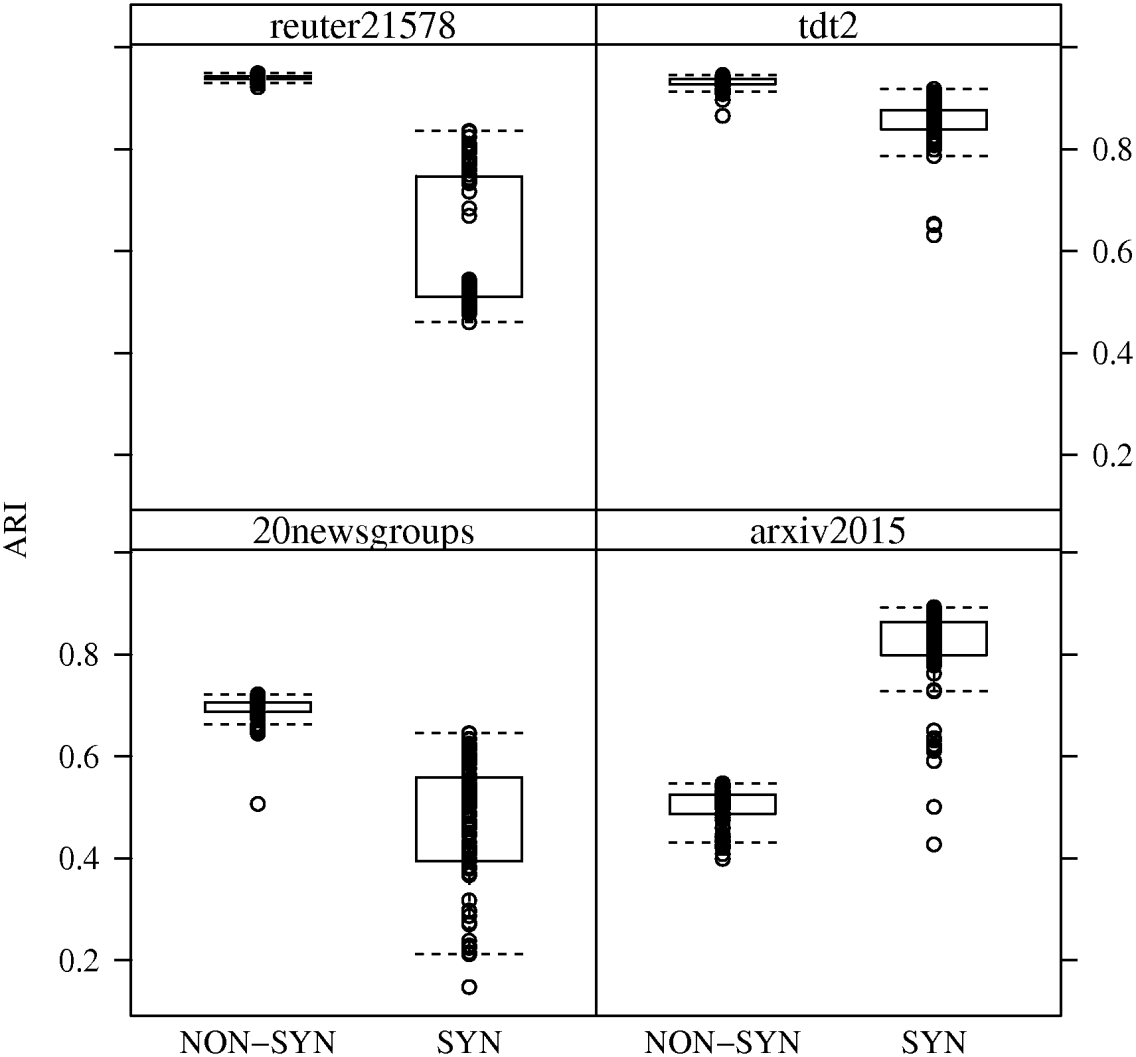
ARI performance of *SOTXTSTREAM* in the presence of concept drift. ARI performance box-plots for *SOTXTSTREAM* with respect to synthetic and non-synthetic random stream orderings. In each run, parameters were set to those listed in Table 2.

**Fig 3:**
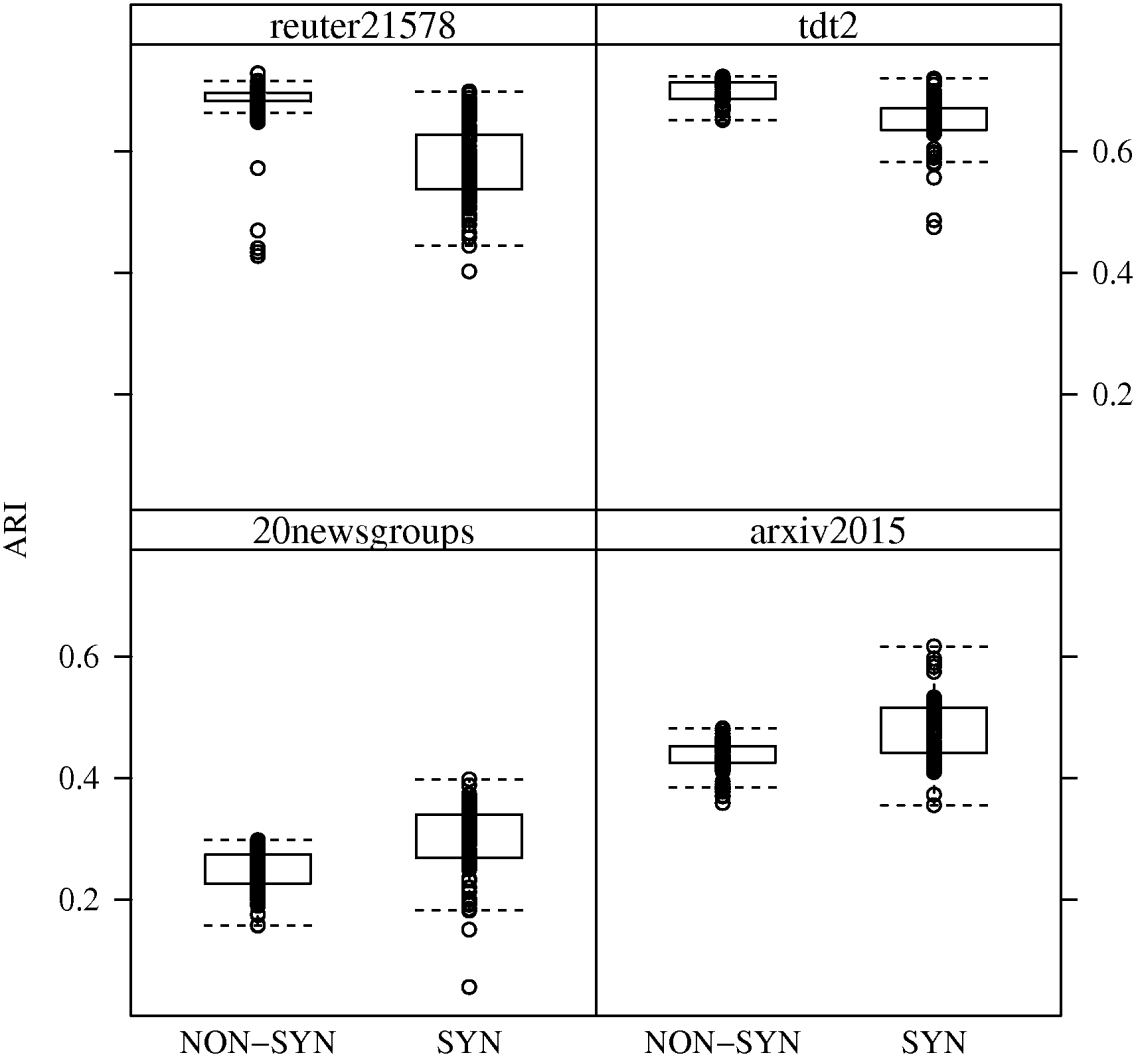
ARI performance of *SOSTREAM* in the presence of concept drift. ARI performance box-plots for *SOSTREAM* with respect to synthetic and non-synthetic random stream orderings. In each run, parameters were set to those listed in Table 2.

By comparing ARI performance of the algorithms with respect to synthetic versus non-synthetic datasets, one can observe the impact of concept drift. In most cases, performance decreases, in varying degrees, with the presence of concept drift. An interesting case is the *arxiv2015* dataset where ARI performance actually increases across all streaming algorithms. The reason for these changes in ARI performance can be observed in Figs 2 and 3, which show boxplots of ARI performance for *SOTXTSTREAM* and *SOSTREAM* in the presence of concept drift. Namely, the variance in ARI performance for the randomly generated stream orderings is greater with concept drift.

In fact one might conclude that performance of *SOSTREAM* is less effected by concept drift, though with an overall lower average performance. However, this difference in variance is most likely attributed to the number of micro-clusters produced by the two algorithms. Also, this may primarily speak to the robustness of the selected parameters with respect to concept drift. In particular, as parameters were optimized with respect to average performance over all random orderings.

### Parameter analysis

**Fig 4:**
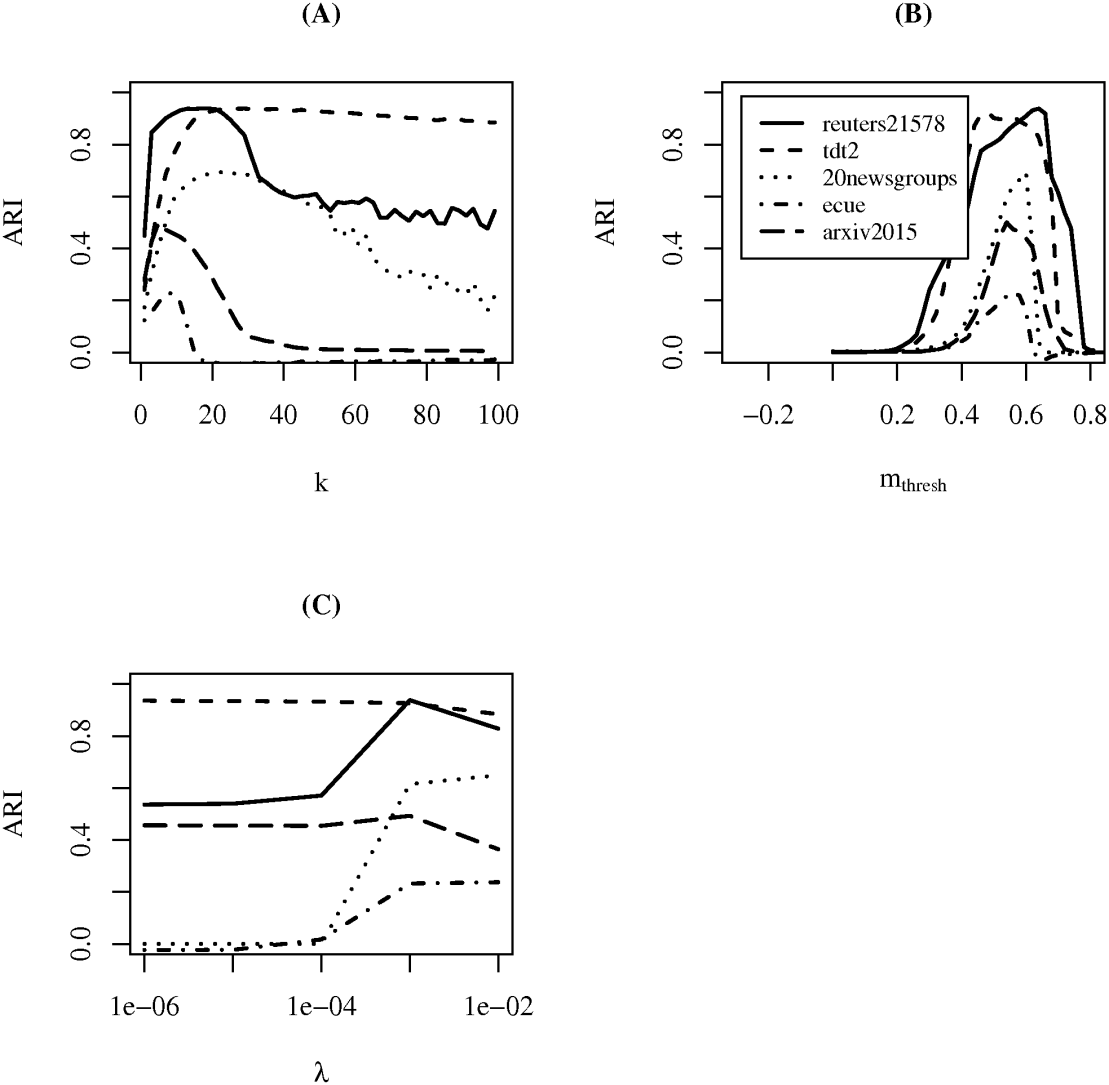
Parameter analysis of *SOTXTSTREAM*. ARI performance plots for *SOTXTSTREAM* algorithm parameters (*k* **(A)**, *m*_*thresh*_ **(B)**, *λ* **(C)**) on all datasets. In each run, parameters were set to those listed in Table 2 (sans the parameter under investigation). Additionally, ARI performance is the average value across 100 random stream orderings.

In Fig 4, the ARI performance of *SOTXTSTREAM* versus values for parameters *k*, *m*_*thresh*_, and *λ* are plotted. With respect to the choice of *k*, Fig 4A, for all datasets, optimal performance is observed at relatively small values of *k* with respect to the specified range. Additionally, in all cases, a decrease in ARI performance is observable following some clear change point (peak or elbow). Furthermore, the rate of decrease following the change point appears to be dataset dependent. Fortunately, an acceptable default value of *k* is observed around *k* = 10 (i.e., near maximum performance for all datasets).

For the choice of the *λ* parameter, Fig 4C, the performance of each dataset is optimal at the same point. Unsurprisingly, as for all datasets, this point is at the constant chosen for each dataset as a function of its size (i.e., parameter optimization was performed at this value). Notwithstanding the aforementioned bias, in some cases poor performance is observed as *λ* approaches zero (at which point no fading is performed). Note that in practice, larger values of *λ*, will result in vectors being faded to 0. In order to avoid this from happening, the largest value of *λ* considered here is 0.01. Additionally, this situation can be avoided completely through the periodic removal of aging clusters.

In the case of the *m*_*thresh*_ parameter, Fig 4B, performance of each dataset is optimal within the [0.5–0.6] range. This appear to be a good threshold in general given text documents, and the use of *TF-IDF* weighting and cosine distance (see optimal parameters for *LSTREAM*, *SOSTREAM*, and *SOTXTSTREAM* in Table 2). As with the choice of *k*, these results suggest the existence of a reasonable default value for the *m*_*thresh*_ parameter.

**Fig 5:**
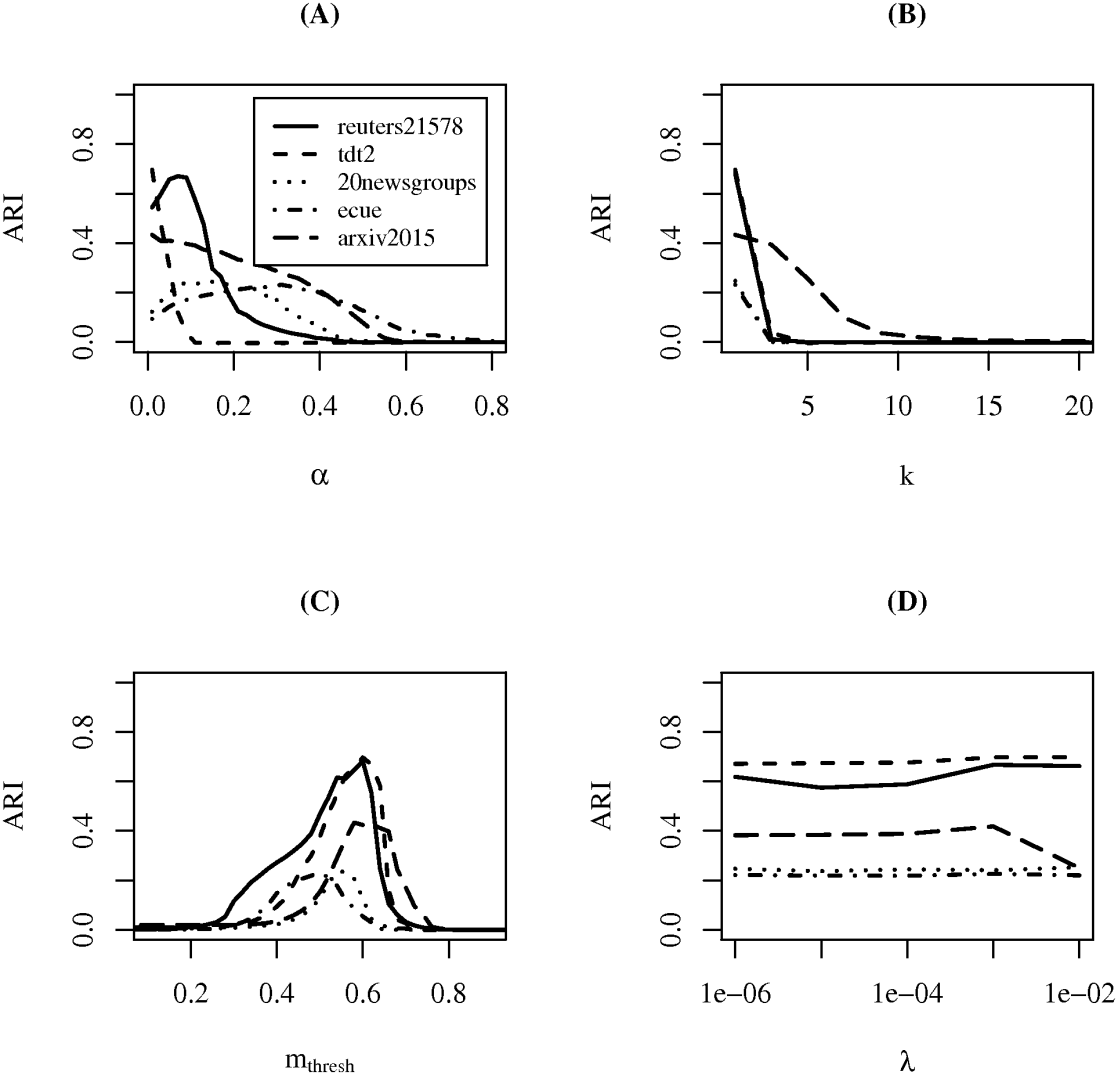
Parameter analysis of *SOSTREAM*. ARI performance plots for the *SOSTREAM* algorithm parameters (*α* **(A)**, *k* **(B)**, *m*_*thresh*_ **(C)**, *λ* **(D)**) on all datasets. In each run, parameters were set to those listed in Table 2 (sans the parameter under investigation). Additionally, ARI performance is the average value across 100 random stream orderings.

Finally, in Fig 5 ARI performance of *SOSTREAM* versus values for parameters *α*, *k*, *m*_*thresh*_, and *λ* are plotted. With respect to *α*, Fig 5A, the optimal choice of *α* appears to be dataset dependent, though performance does converge to zero as *α* approaches one. Additionally, a good default value is observable at *α* = 0. However, at *α* = 0 the self-organizing phase has no effect on results (i.e., learning rate is zero).

For the choice of *k*, Fig 5B, in all datasets performance drops sharply where *k* > 1. In fact, over the course of these experiments it was observed that such cases, *k* > 1, were only viable at *α* = 0. Similarly, note that in all of the cases where *α* > 0 the optimal choice of *k* is one (see Table 2). These last two observations suggest that *k* is highly dependent on *α* and vice versa. This dependence is complicated in *SOSTREAM* as the value *k* is reused in three cluster micro-cluster maintenance operations (insertion, neighborhood adjusting, and merging), whereas only one of these operations (neighborhood adjusting) is dependent on *α*.

As with *SOTXTSTREAM*, performance is optimal for all datasets within the [0.5–0.6] range of the *m*_*thresh*_ parameter, Fig 5C. Additionally, recall the optional use of *m*_*thresh*_ in *SOSTREAM*, as a merge criterion of overlapping micro-cluster radii is applied. Performance of this option is seen here where *m*_*thresh*_ = 1, and it is decidedly poor. Lastly, for the *λ* parameter, Fig 5D, performance seems to be unaffected by the choice of this value. This supports the previous assertion with *SOTXTSTREAM*. In particular, that variability in performance over *λ* is primarily due to its use in the self-organizing phase. However, as all of the datasets are randomly ordered, it’s difficult to draw conclusions with respect to the effect of the fading parameter *λ* on performance.

## Conclusion

A new density-based self-organizing text stream clustering algorithm *SOTXTSTREAM* was presented, and shown to perform better than the *SOSTREAM* algorithm (the sole prior approach to density-based self-organizing stream clustering) on several real-world text streams. This improved performance was achieved by addressing several shortcomings of SOSTREAM. Specifically, this involved removing the use of a fixed learning rate, and decoupling the dependence of three cluster maintenance phases (insertion, adjusting, and merging) on a single neighborhood size parameter. This had the added benefit of eliminating the high dependence the fixed learning rate has on the choice of the neighborhood size parameter in *SOSTREAM*. Likewise, *SOTXTSTREAM* was shown superior, in several cases, and competitive, in the remaining cases, to a popular non-streaming clustering approach. This comparison is significant as *SOTXTSTREAM* is limited to a single pass over the data.

In addition to improving performance, *SOTXTSTREAM* is dependent on two parameters (*k* and *m*_*thresh*_), as compared to *SOSTREAM*’s three (*k*, *m*_*thresh*_, and *α*). Note that here the choice of the *λ* parameter, which both algorithms employ, is expected to be made with some degree of domain knowledge with respect to the desired clusterings.

Future work includes investigating insertion criteria for the nearest cluster of a new stream instance, and methods for calculating influence of an instance on neighboring clusters. Also, experiments conducted over the course of this work has shown potential for replacing the fixed *m*_*thresh*_ parameter with a dynamic one (e.g., an online mean *k* distance of instances within a sliding window).
